# Statin use and survival outcomes in endocrine-related gynecologic cancers: A systematic review and meta-analysis

**DOI:** 10.18632/oncotarget.17242

**Published:** 2017-04-19

**Authors:** Weimin Xie, Li Ning, Yuenan Huang, Yan Liu, Wen Zhang, Yingchao Hu, Jinghe Lang, Jiaxin Yang

**Affiliations:** ^1^ Department of Obstetrics and Gynecology, Peking Union Medical College Hospital, Chinese Academy of Medical Sciences and Peking Union Medical College, Beijing, China; ^2^ Department of General Surgery, The Second Affiliated Hospital, Harbin Medical University, Harbin, China; ^3^ Department of Epidemiology and Statistics, Institute of Basic Medical Sciences, Chinese Academy of Medical Sciences, School of Basic Medicine, Peking Union Medical College, Beijing, China

**Keywords:** gynecologic cancer, statin, survival outcomes, meta-analysis

## Abstract

Previous studies investigating the association between statin use and survival outcomes in gynecologic cancers have yielded controversial results. We conducted a systematic review and meta-analysis to evaluate the association based on available evidence. We searched the databases of the Cochrane Central Register of Controlled Trials (CENTRAL), Embase, and PubMed from inception to January 2017. Studies that evaluated the association between statin use and survival outcomes in gynecologic cancers were included. Pooled hazard ratios (HRs) for overall survival, disease-specific survival and progression-free survival were calculated using a fixed-effects model. A total of 11 studies involving more than 6,920 patients with endocrine-related gynecologic cancers were identified. In a meta-analysis of 7 studies involving 5,449 patients with endocrine-related gynecologic cancers, statin use was linked to improved overall survival (HR, 0.71; 95% confidence interval [CI], 0.63 to 0.80) without significant heterogeneity (*I*^2^ = 33.3%). Statin users also had improved disease-specific survival (3 studies, HR, 0.72; 95% CI, 0.58 to 0.90, *I*^2^ = 35.1%) and progression-free survival (3 studies, HR, 0.68; 95% CI, 0.49 to 0.93, *I*^2^ = 33.6%) in endocrine-related gynecologic cancers. Our findings support that statin use has potential survival benefits for patients with endocrine-related gynecologic cancers. Further large-scale prospective studies are required to validate our findings.

## INTRODUCTION

Gynecologic cancers are a group of malignancies of the female genital system, including ovarian, endometrial, cervical, vaginal, and vulvar cancer. In 2016, an estimated 105,890 new cases of gynecologic cancers and 30,890 gynecologic cancer-related deaths occurred in the United States alone [1]. Among the gynecologic cancers, endometrial and ovarian cancers are considered endocrine-related cancers because they are influenced by hormonal and reproductive events. Ovarian cancer is the deadliest type of gynecologic cancer, with an overall 5-year survival rate of roughly 30–40% [2]. Endometrial cancer is the most common gynecologic cancer in developed countries. Although most women (75%) are diagnosed at an early stage, patients with advanced disease still have a poor prognosis [3]. Despite the advent of molecular targeted drugs and advancements in surgical procedures, the overall prognosis of gynecologic cancers remains grave [4]. Therefore, it is imperative to identify relevant prognostic factors in order to improve the prognosis of gynecologic cancers.

Statins are a group of commonly prescribed medications used primarily for the management of hypercholesterolemia and prevention of coronary heart disease [5–6]. They block 3-hydroxy-3-methyl-glutaryl-coenzyme A (HMG-CoA) reductase, the rate-limiting enzyme for conversion of HMG-CoA to the cholesterol precursor mevalonic acid [7]. The inhibition of the mevalonic acid pathway leads to critical changes in cellular functions. Interestingly, preclinical studies have found that statins also have antineoplastic potential through the induction of tumor cell apoptosis and inhibition of tumor cell proliferation, invasion, and migration [8–10]. These effects have also been shown in ovarian and endometrial cancer-derived cell lines [11–12]. Indeed, a body of epidemiologic studies has demonstrated that statins are associated with improved survival outcomes in several malignancies, including breast, gastric, colorectal, prostate, and kidney cancer [13–17].

A number of studies have evaluated the relationship between statin use and survival outcomes in gynecologic cancers; however, the findings are inconsistent. Therefore, we performed a systematic review of the available evidence, in order to determine whether statin use was in fact associated with improved survival outcomes in patients with gynecologic cancers.

## RESULTS

### Study selection

**Figure 1:**
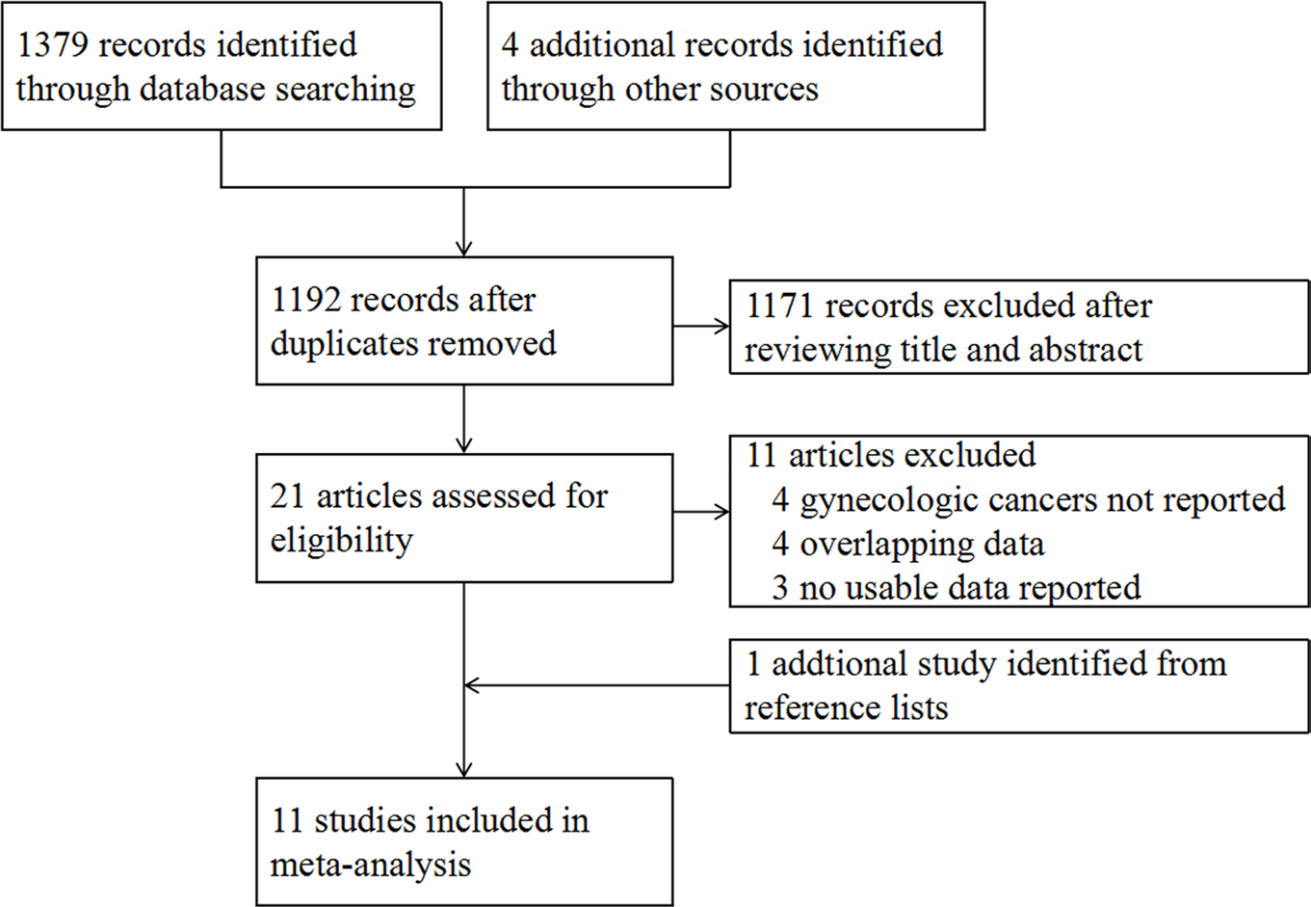
Study flow diagram

In our initial search, we identified 1,379 records from the database search and 4 published abstracts from conference proceedings. After screening the titles and abstracts, 21 potentially relevant records were retrieved for further review. Of these, we excluded 11 studies for the following reasons: 4 did not report the survival outcomes in gynecologic cancers [18–21], 4 used overlapping data [22–25], and 3 did not have usable data [26–28]. We identified no additional ongoing trials from trial registers. One study was retrieved from reference lists [29]. Finally, 11 studies that met our eligibility criteria were included in the meta-analysis. The flow diagram summarizing the process of study selection is shown in Figure 1.

### Study characteristics

A total of 11 non-randomized studies involving more than 6,920 patients with endocrine-related gynecologic cancers were included in the meta-analysis, with 10 cohort studies [29, 31–39] and 1 case-control study [30]. Two of the 11 studies were published only in abstract form [35, 39]. The studies were all published between 2008 and 2016. Of these, 8 studies were carried out in the United States [29, 31–36, 39], 2 in Israel [30, 38], and 1 in China [37]. The effects of statins on mortality and progression in endometrial cancer [29–33] and ovarian cancer [29, 31, 34–39] were evaluated in 5 and 8 studies, respectively. The Newcastle–Ottawa scale values ranged from 4 to 8 stars: 1 study was awarded 4 stars, 2 studies were award 6 stars, and 6 studies were award 7 or more stars. The characteristics of the included studies are shown in Table 1.

**Table 1:** Characteristics of included studies

First author	Study location	Study design	Type of cancer	Stage	Grade	Primary treatment(s)	No. of patients	No. of patients on statins	Statin exposure	Outcomes of interest	Adjusting factors*	NOS value
Lavie et al., 2013	Israel	Case–control	EC	NA	NA	NA	274	45	Post-diagnosis use	OS	1	7
			OC	NA	NA	NA	150	16				
Nevadunsky et al., 2015	USA	Retrospective cohort	EC	I–IV	1–3	NA	983	220	NA	DSS	NA	4
Yoon et al., 2015	USA	Retrospective cohort	EC	I–IV	1–3	Hysterectomy ± radiotherapy ± chemotherapy	2,987	1,893	Post-diagnosis use	OS	1–14	8
Feng et al., 2016	USA	Retrospective cohort	high-grade EC	I–IV	NA	Surgery ± radiotherapy ± chemotherapy	199	50	NA	OS, PFS	1, 2, 4, 16, 17, 18, 12–15, 19–22	6
Wang et al., 2016	USA	Prospective cohort	OC	NA	NA	NA	NA	NA	Current user (at the time of the latest medication inventory)	DSS	1, 2, 13, 18, 20, 23, 24–33	7
			EC	NA	NA	NA	NA	NA				
Elmore et al., 2008	USA	Retrospective cohort	OC	III–IV	3 (93%)	CRS + platinum-based chemotherapy	126	17	Post-diagnosis use	OS, PFS	1, 4, 5, 34	6
Amsler et al., 2013	USA	Retrospective cohort	OC	NA	NA	NA	46	21	NA	RFS	1, 4, 16, 35	–
Habis et al., 2014	USA	Retrospective cohort	OC	I–IV	1–3	CRS + platinum-based chemotherapy	96	68	Post-diagnosis use	PFS, DSS	1, 2, 4, 5, 16, 18, 34, 36–39	7
Chen et al., 2016	China	Retrospective cohort	OC	III–IV	1–3	CRS + platinum-based chemotherapy	60	30	Post-diagnosis use	OS	1, 4, 5, 16, 38	7
Bar et al., 2016	Israel	Retrospective cohort	OC	I–IV	NA	CRS + platinum-based chemotherapy	143	43	Post-diagnosis use	OS, RFS	1, 4, 7, 13, 14, 19, 37, 40	8
Vogel et al., 2016	USA	Retrospective cohort	OC	NA	NA	Surgical resection + platinum therapy	1,510	636	Post-diagnosis use	OS	1, 2, 4, 7, 9, 31	–

Abbreviations: EC, endometrial cancer; OC, ovarian cancer; OS, overall survival; DSS, disease-specific survival; PFS, progression-free survival; RFS, recurrence-free survival; NOS, Newcastle-Ottawa scale; CRS, cytoreductive surgery.

* 1, Age at diagnosis; 2, race; 3, neighborhood income; 4, tumor stage; 5, tumor grade; 6, hysterectomy type; 7, chemotherapy; 8, radiation; 9, Charlson score; 10, impaired glucose tolerance; 11, obesity; 12, dyslipidemia; 13, diabetes; 14, hypertension; 15, parity; 16, histology subtype; 17, lymph node involvement; 18, BMI; 19, aspirin use; 20, smoking history; 21, treatment modality; 22, use of nonstatin lipid-lowering medications; 23, education; 24, physical activity; 25, family history of cancer; 26, current health-care provider; 27, oral contraception use; 28, prior unopposed oestrogen use; 29, prior oestrogen plus progestin use; 30, solar irradiance (latitude); 31, prior CHD history; 32, randomization into the CaD trial; 33, age at menarche; 34, primary cytoreductive surgery; 35, comorbidity; 36, American Society of Anesthesiologists (ASA) class; 37, metformin use; 38, residual tumor; 39, tumor site; 40, use of beta-blockers.

### Meta-analysis

### Overall survival

**Figure 2:**
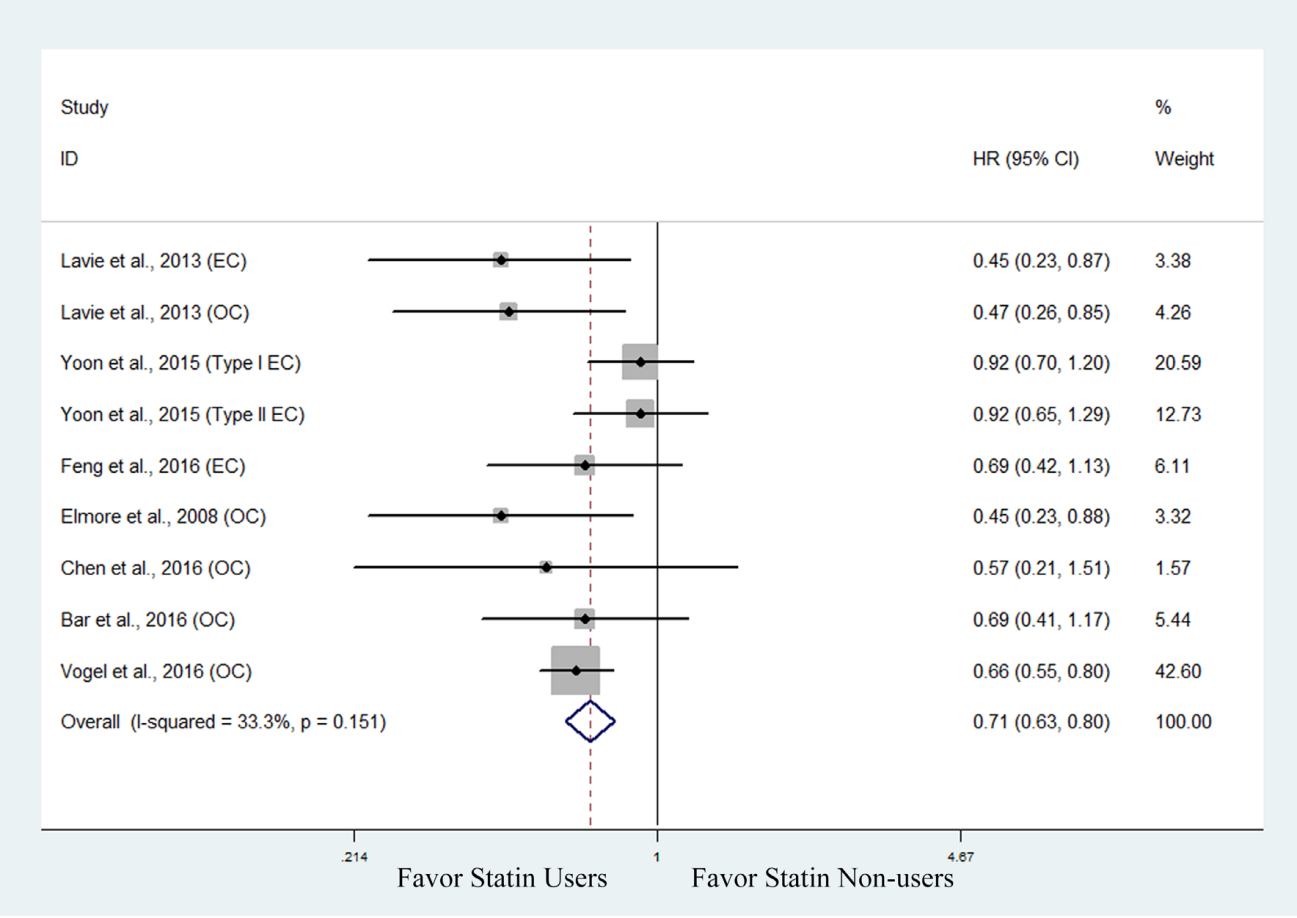
Forest plot of the effect of statin use on overall survival in endocrine-related gynecologic cancer patients

Seven studies involving 5,449 patients with endocrine-related gynecologic cancers investigated the association between statin use and overall survival (OS) [30, 32–34, 37–39]. The pooled data showed that statin use was associated with improved OS (HR, 0.71; 95% CI, 0.63 to 0.80). The Chi-square test resulted in a *p* value of 0.151 and the corresponding *I*^2^ was 33.3%, both indicating no significant heterogeneity (Figure 2).

**Figure 3:**
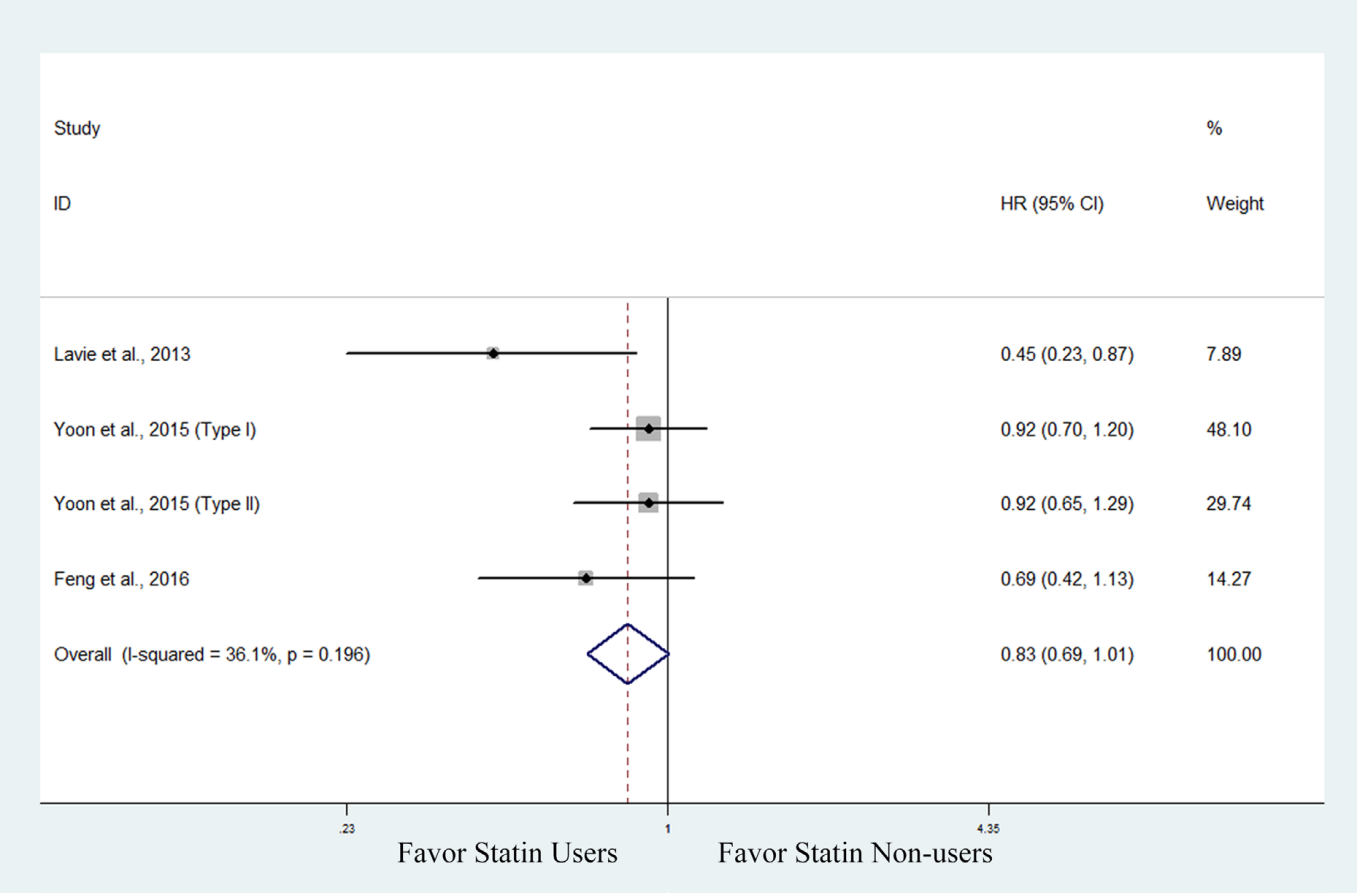
Forest plot of the effect of statin use on overall survival in endometrial cancer patients

**Figure 4:**
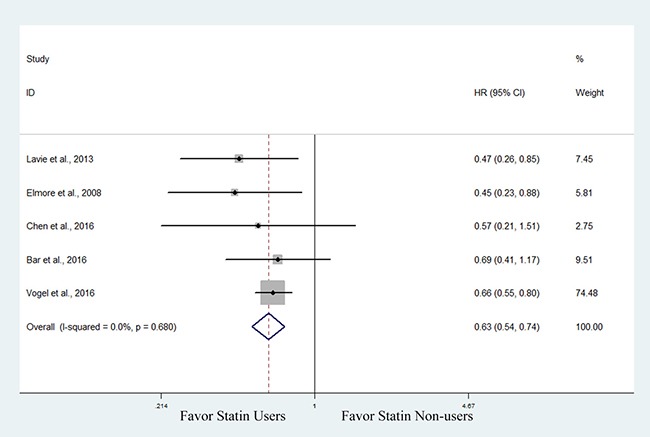
Forest plot of the effect of statin use on overall survival in ovarian cancer patients

Then, we performed a subanalysis based on cancer type. Three studies involving 3,460 patients with endometrial cancer evaluated the association between statin use and OS [30, 32, 33]. The pooled data showed improved OS in statin users, though the data supporting this association was not as robust (HR, 0.83; 95% CI, 0.69 to 1.01) (Figure 3). One of the 3 studies also found that hyperlipidemic patients with endometrial cancer who used statins had improved OS compared with those not using statins (HR, 0.42; 95% CI, 0.20 to 0.87) [33]. Five studies involving 1,989 patients with ovarian cancer evaluated the association between statin use and OS [30, 34, 37–39]. The pooled data showed that statin users had a significantly improved OS compared with non-users (HR, 0.63; 95% CI, 0.54 to 0.74) (Figure 4).

### Disease-specific survival

**Figure 5:**
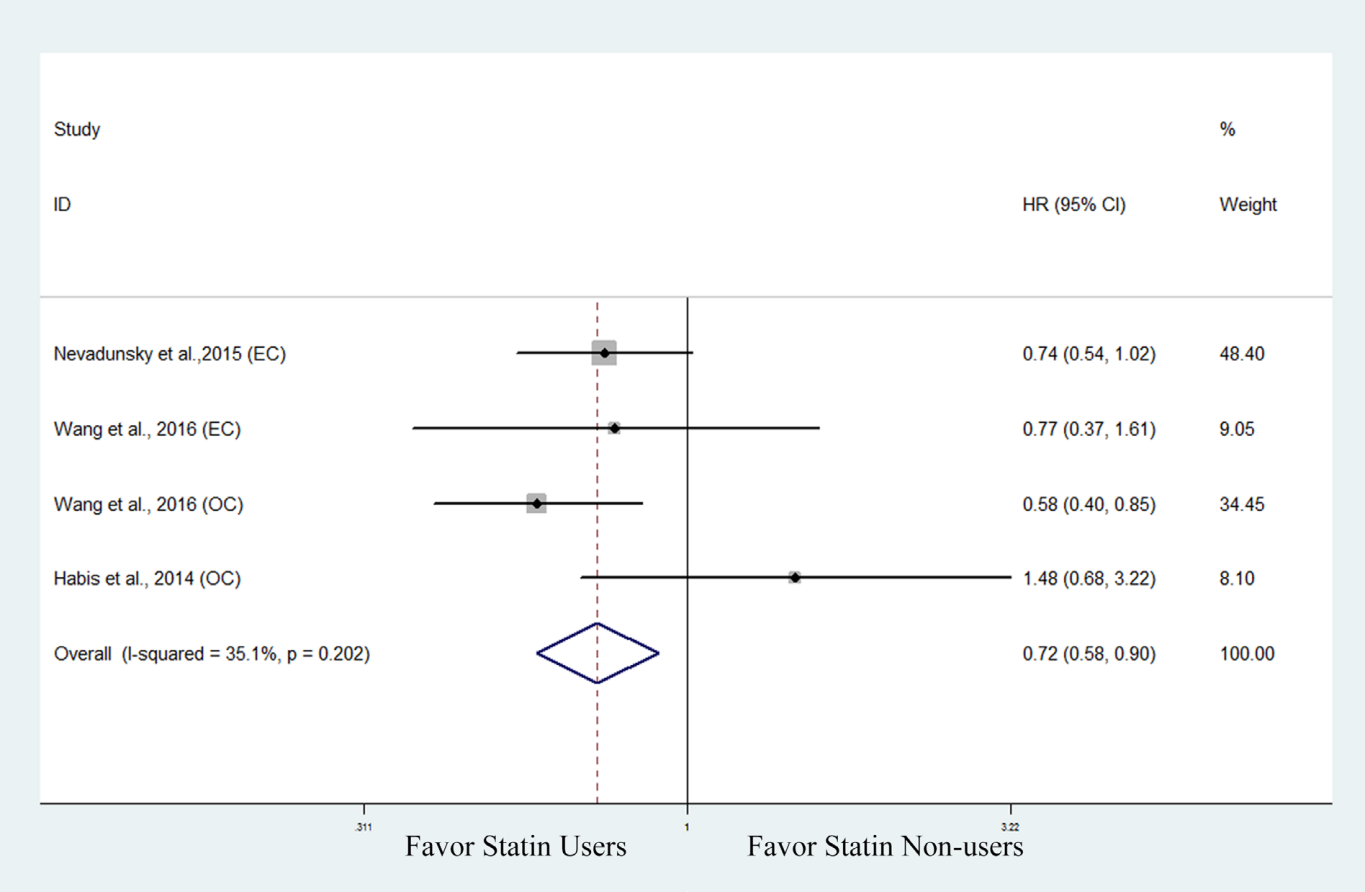
Forest plot of the effect of statin use on disease-specific survival in endocrine-related gynecologic cancer patients

Three studies investigated the association between statin use and disease-specific survival (DSS) in endocrine-related gynecologic cancers [29, 31, 36]. The pooled data showed that statin use was significantly associated with improved DSS (HR, 0.72; 95% CI, 0.58 to 0.90). The Chi-square test resulted in a *p* value of 0.202 and the corresponding *I*^2^ was 35.1%, indicating no significant heterogeneity (Figure 5). We did not perform a subanalysis based on cancer type as only two studies reported on DSS in endometrial cancer [29, 31] and ovarian cancer [29, 36], respectively.

### Progression free survival

**Figure 6:**
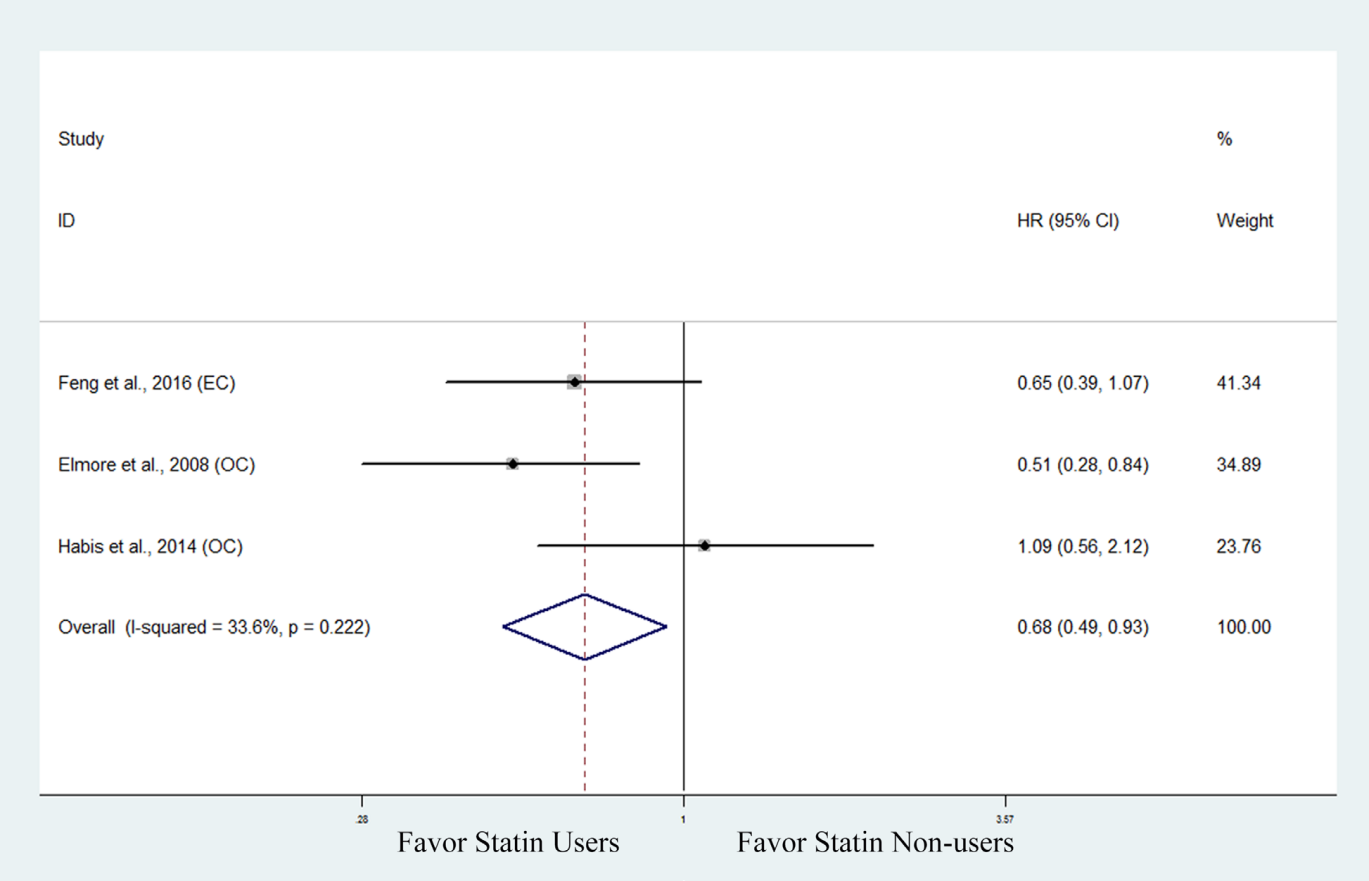
Forest plot of the effect of statin use on progression free survival in endocrine-related gynecologic cancer patients

Three studies involving 421 patients with endocrine-related gynecologic cancers explored the association between statin use and progression-free survival (PFS) [33, 34, 36]. The pooled data showed that statin use was significantly associated with improved PFS (HR, 0.68; 95% CI, 0.49 to 0.93). The Chi-square test resulted in a *p* value of 0.222 and the corresponding *I*^2^ was 33.6%, indicating no significant heterogeneity (Figure 6). We did not perform a subanalysis based on cancer type as PFS in endometrial cancer [33] and ovarian cancer [34, 36] was only reported in 1 and 2 studies, respectively.

### Data not included in the meta-analysis

As the effect of statin use on RFS was only evaluated in two studies involving 189 patients with ovarian cancer [35, 38], a meta-analysis was not performed for RFS. While no association was found between statin use and RFS (HR 0.66, 95% CI, 0.40 to 1.08) in one study [38], statin users had significantly improved RFS (HR 0.17, 95% CI, 0.04 to 0.73) in the other study [35]. Two studies compared the survival outcomes of statin users and statin non-users in a hyperlipidemic cohort [33, 36]. Feng et al. [33] found that hyperlipidemic patients with endometrial cancer who used statins showed improved OS (HR, 0.42; 95% CI, 0.20 to 0.87) and PFS (HR, 0.47; 95% CI, 0.23 to 0.95) compared with those who did not use statins, whereas Habis et al. [36] found that statin use was not significantly associated with PFS (HR, 1.09; 95% CI, 0.56 to 2.12) or DSS (HR, 1.48; 95% CI, 0.68 to 3.22) in hyperlipidemia patients with ovarian cancer.

## DISCUSSION

There is a long-standing debate regarding the association between statin use and survival outcomes in gynecologic cancers. Meta-analysis provides an objective evaluation of the evidence, which may lead to the resolution of uncertainty and controversy by permitting a synthesis of data [40]. In accordance with the promising findings derived from *in vitro* and animal studies [11, 12, 41], the present meta-analysis provide supportive evidence for an association between statin use and improved survival outcomes in endocrine-related gynecologic cancers. In this meta-analysis of 11 non-randomized studies, we found that patients with endocrine-related gynecologic cancers who used statins showed significantly improved OS, DSS, and PFS. Our results are consistent with recent meta-analyses regarding the protective effect of statin use on other site-specific cancers. Similarly, these reports concluded that statin use was associated with improved survival outcomes in colorectal, breast, prostate and kidney cancer [42–45].

In our subanalysis based on cancer type, statin use was generally associated with improved OS in patients with ovarian and endometrial cancers. However, the prediction intervals for endometrial cancer crossed the value of 1 in our meta-analysis. This implies that while statin use is averagely associated with improved OS in endometrial cancer (pooled HRs < 1), there may be certain populations in which statins would not improve the survival. Interestingly, Feng et al. [33] found that statin users had a significantly improved OS compared with non-users in the subset of patients with endometrial cancer and hyperlipidemia. This may support the preclinical findings of statin effects through the mevalonic acid pathway. To better understand this issue, future studies are needed to identify exactly which subgroups of patients with endometrial cancer might benefit from statins.

Proposed mechanisms to explain the protective effect of statins on endocrine-related gynecologic cancers include cholesterol lowering and systematic anti-inflammatory effects through the mevalonic acid pathway [46]. The lowering of cholesterol may reduce metabolites that are crucially involved in cell proliferation, angiogenesis, and migration. Murine models of ovarian cancer were found to undergo decreased cell proliferation and increased apoptosis when treated with statins [41]. Statins also affect the proliferation of tumor cells in murine models of breast, colon, pancreatic, liver, and prostate cancers [47–50]. Additionally, statins can also stimulate inflammatory responses and anticancer immune surveillance via the phosphorylation of Akt and down-regulation of the mammalian target of rapamycin (mTOR) [51].

Generally, a meta-analysis of RCTs is less likely to provide biased results and thus allows for a more objective appraisal of evidence than that of non-randomized studies. However, for those specific questions that cannot be answered by reviews of RCTs, such as limited number of studies, non-randomized studies should be retrieved for meta-analysis. To date, no RCTs have established the association between statin use and survival outcomes in gynecologic cancers. Therefore, we conducted this meta-analysis by pooling the results from 11 non-randomized studies.

The present study has some important strengths. First, a comprehensive, systematic, and reproducible search for relevant published and unpublished papers was performed. No exclusion criteria in terms of language, methodological characteristics or place of publication were applied. Hence, the likelihood of important selection or publication bias in the review process was small. Second, no significant heterogeneity was present in any of the analyses, including OS, DSS, and PFS, which reinforced our confidence in the reliability of the pooled results. Third, most studies included in our meta-analysis had high methodological quality scores, which further enhanced the reliability of our results. To the best of our knowledge, the present study is the first article to investigate the relationship between statin use and survival outcomes in endocrine-related gynecologic cancers.

Still, this meta-analysis has some limitations. First, as there were no relevant RCTs in the literature to date, all of the included studies were non-randomized studies. Second, most of the included studies were retrospective in design; thus, it was impossible to eliminate the possibility of recall bias, and the true effect of the statins might be overestimated due to the lack of experimental random allocation to the intervention. Third, 2 abstracts without available full texts were included in our study, which made it difficult to properly assess their methodological qualities. Fourth, some studies did not provide information regarding tumor stage, tumor grade, primary treatments, or the definition of DSS and PFS, which may have introduced a bias. In addition, the method of adjustment for potential confounding factors was not consistent in all of the studies. Even though the multivariate Cox proportional hazards model was employed in most studies, only univariate analysis was applied in the studies without the necessary data. Therefore, our results should be interpreted cautiously, and further prospective randomized trials are required for a more definitive understanding. Finally, the limited number of included studies made it impractical to evaluate the effects of statins according to type, dose, frequency, and duration of use.

In summary, the findings of this systematic review and meta-analysis demonstrate that statin use is potentially beneficial in terms of OS, DSS, and PFS in endocrine-related gynecologic cancers. Since we cannot exclude the potential methodological limitations of each individual study, biases of these findings may have been introduced and these results should be interpreted with caution. The full potential roles of statins in endocrine-related gynecologic cancers should be evaluated further in large-scale prospective studies.

## MATERIALS AND METHODS

This meta-analysis was prepared according to the guidelines proposed by the Meta-Analysis of Observational Studies in Epidemiology (MOOSE) group [52].

### Search strategy

We performed a systematic search using the databases of the Cochrane Central Register of Controlled Trials (CENTRAL), Embase, and PubMed to find all relevant articles from inception to January 2017. Both subject headings and free text words were used in the search. The detailed search strategies are presented in Appendix A. We searched the following trial registers electronically for potentially relevant ongoing trials: ClinicalTrials.gov (https://clinicaltrials.gov/), World Health Organization International Clinical Trials Registry Platform (ICTRP) (http://apps.who.int/trialsearch/), and ISRCTN registry (http://www.isrctn.com/mrct/). We also searched for conference reports from 2008 to 2016 by hand searching and electronic searching in the following sources: Biennial Meeting of the International Journal of Gynecological Cancer Society (IGCS), Biennial Meeting of the European Society of Gynecological Oncology (ESGO), Annual Meeting of the American Society of Clinical Oncology (ASCO), and Annual Meeting on Women's Cancer of the Society of Gynecologic Oncology (SGO). In addition, we screened the reference lists of all of the retrieved articles for additional eligible studies. No language restriction was applied in our search strategy.

### Eligibility criteria

After conducting the search, 2 reviewers (W. X. and L. N.) removed duplicate records and screened the titles and abstracts independently. The potentially relevant references were evaluated in detail to determine their eligibility. Studies were considered in this meta-analysis if they met the following inclusion criteria: (1) randomized controlled trials (RCTs) or non-randomized studies; (2) evaluated the association between statin use and survival outcomes in gynecologic cancers; (3) evaluated at least 1 of the outcomes of interest, including overall survival (OS), disease-specific survival (DSS), progression free survival (PFS), and recurrence-free survival (RFS); (4) reported hazard ratio (HR) and a 95% confidence interval (CI), or provided data for their calculation. Articles were excluded if they were: (1) editorials, letters, reviews, and case reports; (2) studies without appropriate data that could be extracted or calculated. In cases of duplicate publications involving the same population, only the most comprehensive studies were included. Any disagreements in study selection were resolved by discussion between the 2 reviewers and, if needed, in consultation with a third reviewer (Y. H.).

### Data extraction and quality assessment

Two reviewers (W. X. and L. N.) extracted data independently. The following data were collected from each study: publication data (i.e., the first author's name, publication year, and study location), study design, publication type, type of cancer, sample size, definition of statin exposure, follow-up, HR and 95% CI, and adjusting factors. When multiple estimates of effect (HR) were presented, the most adjusted estimate was extracted; when an adjusted estimate was not available, the crude estimate was extracted. When the HR and 95% CI were not available, we estimated them indirectly from Kaplan-Meier curves using published methods [53, 54].

Three reviewers (W. X., L. N. and Y. H.) evaluated the methodological quality of the included studies independently. Since all of the included studies were non-randomized studies, their quality was assessed using the Newcastle–Ottawa scale [55], which uses a star system ranging from 0 to 9 stars. Studies that were awarded 7 or more stars were considered of high quality.

### Statistical analysis

The HRs from each of the individual eligible studies were combined to form a pooled HR. Heterogeneity was measured using the Chi-square (χ^2^, or Chi^2^) and *I*^2^ tests. When significant heterogeneity (*p* value < 0.10 or *I*^2^ > 50 %) was found, a random-effects model was applied to calculate the pooled effect; otherwise, a fixed-effects model was used. Given the limited number of studies in the meta-analysis, we did not evaluate publication bias [56, 57]. All analyses were performed using Stata version 12.0 software (Stata Corporation, College Station, TX). For all tests, a two-sided *p* value less than 0.05 was considered statistically significant.

## SUPPLEMENTARY MATERIALS FIGURES AND TABLES


