# Supplementary material for: Statin use and survival outcomes in endocrine-related gynecologic cancers: A systematic review and meta-analysis
Source: Oncotarget. 2017 Apr 19;8(25):41508–17. doi: 10.18632/oncotarget.17242 (PMC5522329; doi:10.18632/oncotarget.17242)
Supplement: Supplementary file 1 [file oncotarget-08-41508-s001.pdf]

Statin use and survival outcomes in endocrine-related gynecologic cancers: A systematic review and meta-analysis

Supplementary Materials

Search strategy

Pubmed, Cochrane Central Register of Controlled Trials (CENTRAL) and Embase were searched from the inception to January 14, 2017, using the following search strategy.

Pubmed search strategy

(("Hydroxymethylglutaryl-CoA Reductase Inhibitors"[Pharmacological Action]) OR ("Hydroxymethylglutaryl-CoA Reductase Inhibitors"[Mesh]) OR ("hydroxymethylglutaryl-coa reductase inhibitor*") OR ("HMG-CoA reductase inhibitor*") OR (statin*) OR (atorvastatin*) OR (lovastatin*) OR (mevastatin*) OR (cerivastatin*) OR (fluvastatin*) OR (pitavastatin*) OR (rosuvastatin*) OR (pravastatin*) OR (rivastatin*) OR (simvastatin*)) AND (("Endometrial Neoplasms"[Mesh]) OR ("Uterine Neoplasms"[Mesh]) OR ("Uterine Cervical Neoplasms"[Mesh]) OR ("Ovarian Neoplasms"[Mesh]) OR ("Vaginal Neoplasms"[Mesh]) OR ("Fallopian Tube Neoplasms"[Mesh]) OR ("Vulvar Neoplasms"[Mesh]) OR (((((((((endometr*) OR uter*) OR cervi*) OR ovar*) OR vagin*) OR fallopian) OR vulva*) OR gynae*) OR gyne*) AND ((((((neoplas*) OR malignan*) OR carcinom*) OR tumor*) OR cancer*) OR tumour*)))

CENTRAL search strategy

#1 MeSH descriptor: [Hydroxymethylglutaryl-CoA Reductase Inhibitors] explode all trees
#2 "hydroxymethylglutaryl-CoA reductase inhibitor*" in Trials
#3 "HMG-CoA reductase inhibitor*" in Trials
#4 "HMG-CoA*" or "HMG CoA*" or statin*
#4 "HMG-CoA*" OR "HMG CoA*" OR statin* in Trials
#5 atorvastatin* or lovastatin* or cerivastatin* or fluvastatin* or mevastatin* or rivastatin* or pitavastatin* or pravastatin* or rosuvastatin* or simvastatin* in Trials
#6 #1 or #2 or #3 or #4 or #5
#7 MeSH descriptor: [Endometrial Neoplasms] explode all trees
#8 MeSH descriptor: [Uterine Neoplasms] explode all trees
#9 MeSH descriptor: [Uterine Cervical Neoplasms] explode all trees
#10 MeSH descriptor: [Ovarian Neoplasms] explode all trees
#11 MeSH descriptor: [Vaginal Neoplasms] explode all trees
#12 MeSH descriptor: [Fallopian Tube Neoplasms] explode all trees
#13 MeSH descriptor: [Vulvar Neoplasms] explode all trees
#14 (endometr* or uter* or cervi* or ovar* or vagin* or fallopian* or vulva* or gynae* or gyne*) near/5 (cancer* or neoplas* or carcinom* or malignan* or tumor* or tumour*) in Trials
#15 #7 or #8 or #9 or #10 or #11 or #12 or #13 or #14
#16 #6 and #15

Embase search strategy:

#1 'hydroxymethylglutaryl coenzyme a reductase inhibitor'/exp
#2 'hydroxymethylglutaryl coenzyme a reductase inhibitor*'
#3 'hmg-coa reductase inhibitor*' OR statin*
#4 atorvastatin* OR lovastatin* OR cerivastatin* OR fluvastatin* OR pravastatin* OR mevastatin* OR pitavastatin* OR rosuvastatin* OR rivastatin* OR simvastatin*
#5 #1 OR #2 OR #3 OR #4
#6 'endometrium tumor'/exp
#7 'uterus cancer'/exp
#8 'uterine cervix tumor'/exp
#9 'ovary cancer'/exp
#10 'vagina tumor'/exp
#11 'uterine tube tumor'/exp
#12 'vulva tumor'/exp
#13 endometr* OR uter* OR cervi* OR ovar* OR vagin* OR fallopian OR vulva* OR gynae* OR gyne*
#14 neoplas* OR cancer* OR carcinom* OR malignan* OR tumor* OR tumour*
#15 #13 AND #14
#16 #6 OR #7 OR #8 OR #9 OR #10 OR #11 OR #12 OR #15
#17 #5 AND #16
